# Validation of the Slovenian version of the Montreal Cognitive Assessment Scale as a screening tool for the detection of mild cognitive impairment

**DOI:** 10.1007/s13760-024-02487-z

**Published:** 2024-03-04

**Authors:** Andreja Špeh, Irena Kalar, Zvezdan Pirtošek, Milica Gregorič Kramberger

**Affiliations:** 1 Department of Neurology, University Medical Center Ljubljana, Zaloška Cesta 2a, 1000 Ljubljana, Slovenia; 2 Medical Faculty, University of Ljubljana, Ljubljana, Slovenia; 3 Department of Neurobiology, Care Sciences and Society (NVS), Division of Clinical Geriatrics, Karolinska Institutet, Huddinge, Sweden

**Keywords:** Mild cognitive impairment, MoCA, Screening, Cognition, Elderly

## Abstract

**Objective:**

The Montreal cognitive assessment scale (MoCA) is commonly used for detecting individuals with mild cognitive impairment (MCI). The aim of the present study was to evaluate the validity of the Slovenian MoCA as a screening tool for MCI and to determine the optimal cut-off point to detect MCI in the elderly population**.**

**Methods:**

Mini-Mental State Examination (MMSE), MoCA, and neuropsychological testing assessment were conducted on 93 individuals aged ≥ 60 years. MCI was found in 35 individuals with 58 cognitively asymptomatic controls. Cut-off values, sensitivity, and specificity of MoCA were calculated with the receiver operating characteristic curve.

**Results:**

MCI and healthy individuals did not differ with respect to age and education. Healthy individuals (*M* = 24.5, SD = 1.7) performed significantly better on MoCA compared to MCI individuals (*M* = 21.4, SD = 3.2) (*p* < 0.001). The Cronbach’s α of MoCA as an index of internal consistency was 0.64. MoCA distinguished between healthy controls and MCI individuals with a sensitivity of 77% and specificity of 74%, using a cut-off of 23/24 points.

**Conclusion:**

The Slovenian version of MoCA demonstrates an optimal cut-off value of 23/24 points for detecting older individuals with MCI. As a screening tool for MCI, its better diagnostic accuracy makes it preferable to using MMSE.

## Introduction

Mild cognitive impairment (MCI) is characterised by objective cognitive deficits with mainly preserved functional activities and not meeting criteria for clinically probable dementia [1]. The risk of progression from MCI to dementia has been estimated to range from 6.0% to 44.8% [2]. It is important to realize that MCI is a clinical diagnosis as are the diagnoses of dementia or AD [3]. For the diagnosis, the following criteria, proposed by Petersen [3] should be satisfied: (a) cognitive complaint, preferably corroborated by an informant; (b) objective memory impairment for age; (c) relatively preserved general cognition for age; (d) intact activities of daily living; and (e) not demented. Although, currently no medications or other treatment options are approved specifically for MCI, early diagnosis still offers some benefits; the patient’s future care needs can be to some degree anticipated and adequate arrangements can be made in time, with the patient being involved in these decisions [4].

Although the mini-Mental State Examination (MMSE) [5] is the most commonly used cognitive screening test for MCI and dementia, The Montreal Cognitive Assessment (MoCA) [6] has shown superior diagnostic accuracy for MCI compared to MMSE [4, 7]. MoCA assesses multiple cognitive domains, including attention, concentration, executive functions, memory, language, visuospatial skills, abstraction, calculation, and orientation. The initial validation of the scale with a cut-off score of ≥ 26 reached a sensitivity of 78% and specificity of 90% for the diagnosis of MCI [6]. However, the cut-off values reported by other studies have varied [8–10]. A meta-analysis revealed that a cut-off score of 23, rather than the initially recommended score of 26, lowers the rate of false positives and shows overall better diagnostic accuracy [11].

The aims of the present study are to evaluate the validity of the already standardized and validated MoCA for the Slovenian population as a screening tool for MCI and to determine the optimal cut-off point to detect MCI in the elderly memory clinic population with mild cognitive complaints (and not meeting the criteria for dementia).

## Methods

### Population

Participants were consecutively included from the memory clinic during the years 2016–2019. All participants were ≥ 60 years old and underwent neurological examination, followed by a neuropsychological assessment. The neurological examination included screening tests (MMSE and MoCA), blood tests, brain imaging (computed tomography or magnetic resonance), and, optionally, cerebrospinal fluid (CSF) biomarkers (amyloid β protein fragment 1–42, total-tau, and phosphorylated-tau) testing.

We excluded participants with probable dementia according to the diagnostic criteria [12], history of stroke, acute diseases (cancer and infectious diseases), psychiatric disorders (depression, schizophrenia, bipolar disorder), individuals with missing data on MMSE, MoCA, and neuropsychological battery, and those with 6 months or more in-between the neurological examination and neuropsychological testing session. The final sample included 93 individuals, who were further categorised into MCI and the control group. MCI was diagnosed based on conventional criteria suggested by Peterson and others [3] which relies on impairment on a single neuropsychological test. MCI was defined as an individual’s score ≤ 1.5 SD for their age group on at least one of the three assessed cognitive domains: delayed memory, visuospatial abilities, and executive function.

### Compliance with ethical standards

This study complies with the Declaration of Helsinki and was approved by the national ethical review board in Slovenia (number 44/03/11). The data were de-identified before analysis.

### Cognitive evaluation

Both groups underwent cognitive evaluation at the neurological examination using Slovenian versions of MMSE [13] and MoCA. For participants with 12 years of education or less, one point was added to their total score on the MoCA (if < 30). Within 6 months, all the participants had neuropsychological testing where delayed memory and visuospatial abilities were assessed using Repeatable Battery for the Assessment of Neuropsychological Status (RBANS), and executive function was assessed with the Tower of London test (TOL).

### Statistical analysis

Descriptive statistics were used for the sample’s characterization, and chi-square and two-sample *t*-tests allowed comparisons between the groups. To assess the internal consistency of MoCA, Cronbach’s α was calculated. Cut-off values, sensitivity, specificity, and likelihood ratio of the MoCA for predicting MCI compared to normal aging were determined. As a measure of the predictive value of the test, the area under the curve (AUC) and receiver operating characteristics (ROC) curve were calculated. All statistical analyses mentioned above were performed using STATA 16. The DeLong test [14] was used for comparing the areas under ROC curves of MMSE and MoCA. The test was implemented using the “pROC” package in R (version 4.2.2).

## Results

The characteristics of the study sample, and the two subgroups, are provided in Table 1.

**Table 1:** Demographic data, MMSE, and MoCA results for the total sample, MCI, and healthy controls

	total sample (*n* = 93)	MCI (*n* = 35)	healthy controls (*n* = 58)	*p* value
gender (male), N (%)	44.0 (47.3)	26 (44.8)	18 (51.4)	0.537
age, M (SD)	74.0 (6.6)	74.1 (6.1)	73.9 (7.5)	0.894
years of education, M (SD)	11.8 (3.3)	11.8 (3.2)	11.8 (3.5)	0.943
MMSE, M (SD)	27.0 (2.3)	26.4 (2.5)	27.9 (1.5)	0.001
MoCA, M (SD)	22.6 (3.2)	21.4 (3.2)	24.5 (1.7)	< 0.001
visuospatial, M (SD)	3.6 (1.2)	3.4 (1.3)	4.0 (0.9)	0.016
naming, M (SD)	2.9 (0.4)	2.8 (0.5)	3.0 (0.2)	0.040
attention, M (SD)	5.2 (1.1)	5.0 (1.2)	5.5 (0.7)	0.023
language, M (SD)	1.9 (0.9)	1.7 (0.9)	2.1 (0.8)	0.022
abstraction, M (SD)	1.5 (0.7)	1.4 (0.7)	1.5 (0.7)	0.795
recall, M (SD)	1.4 (1.4)	1 (1.4)	2.1 (1.3)	< 0.001
orientation, M (SD)	4.8 (0.6)	5.7 (0.7)	5.9 (0.3)	0.050

Our sample included 93 individuals, 47.3% of whom were male, with an age range between 60 and 90 (*M* = 74) years. MCI and healthy individuals did not differ with respect to gender, age, and education (Table 1). Compared to individuals with MCI, healthy individuals performed significantly better both on MMSE and MoCA. MCI group achieved lower scores on all domains of the MoCA test, with the exception of abstraction and orientation, for which the difference was borderline significant. In this study, the MoCA scores were positively associated with MMSE scores (*r* = 0.60, *p* < 0.001), while the correlations of MoCA with age (*r* = − 0.18, *r* = 0.09) and years of education (*r* = 0.14, *p* = 0.27) were not significant.

Approximately half of the participants (51.6%) had CSF-testing, most of which had MCI (70.8%), since individuals often decided against CSF-testing or it was not advised to them after having a normal neuropsychological profile. Out of those with MCI, 41.2% had positive CSF markers for AD.

**Fig. 1:**
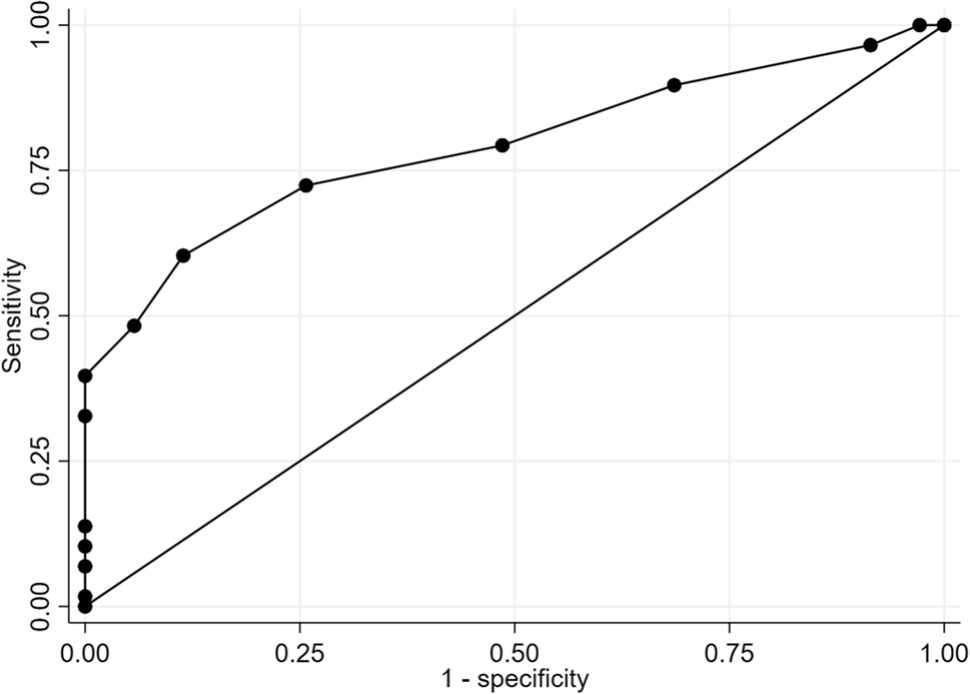
Receiver operator characteristics curve analysis of the MoCA to detect MCI

The Cronbach’α for MoCA was 0.64. The optimal cut-off value for MoCA was 23/24 points (Fig. 1, Table 2). At this value, the area under the curve (AUC) was 0.79 (95% CI 0.70–0.88), sensitivity was 0.77, specificity was 0.74, positive predictive value was 0.82, negative predictive value was 0.62, and the likelihood ratio was 2.96. We have additionally performed ROC analysis for MMSE, which had a lower AUC of 0.68 (CI 0.57–0.79), with a sensitivity of 0.72 and specificity of 0.57 for the cut-off score of 28/29. The difference between AUC of MMSE and MoCA was statistically significant (*z* = − 2.05, *p* = 0.04).

**Table 2:** Cut-off scores, sensitivity, specificity, positive predictive value (PPV), and negative predictive value (NPV) of MoCA for detecting MCI

Value	Sensitivity	Specificity	PPV	NPV
20/21	0.397	1.000	1.00	0.50
21/22	0.483	0.943	0.93	0.52
22/23	0.603	0.886	0.90	0.57
**23/24**	**0.724**	**0.743**	**0.82**	**0.62**
24/25	0.793	0.514	0.73	0.60
25/26	0.897	0.314	0.68	0.65
26/27	0.966	0.086	0.64	0.60
28/29	1.000	0.029	0.63	1.00

## Discussion

The aim of the present study was to evaluate the validity of Slovenian MoCA for detecting MCI. The diagnostic accuracy of 72% for sensitivity and 74% for specificity was determined when a cut-off value of 23/24 was applied (scores of 23 and below indicate impairment). The significant difference in the AUC between MMSE and MoCA provide support for the contention that MoCA is more reliable than the MMSE for the diagnosis of MCI [4, 7], with the AUC for MoCA being 0.79 and for MMSE 0.68. MoCA, compared to MMSE, assesses a wider range of cognitive domains, making it more effective in detecting subtle cognitive changes in a very heterogenous group of individuals, including those with non-amnestic cognitive impairment.

Our results differ from the initial validation of MoCA for the diagnosis of MCI, which suggested a cut-off score of 25/26 with a sensitivity of 90% and specificity of 87% [6], even though our sample was quite similar to theirs in terms of age and education. There have been studies confirming the recommended value [15, 16], however, many studies have shown that a lower cut-off score than the originally recommended had better diagnostic accuracy [17–19]. For example, a longitudinal study revealed that the cut-off score of 26 was too high even for highly educated, cognitively normal older adults [20]. While our study concentrated on the Slovenian population, a comparable examination in a Slavonic cohort, specifically within the Czech sample, found that an optimal sensitivity was achieved with a cut-off score of 24/25 [21]. In line with this, a meta-analysis of 20 studies has shown that a cut-off value of 24/25 correctly diagnosed 80% of patients with MCI [22]. A more recent review and meta-analysis proposed an even lower cut-off of 23/24, which lovers the false positive rate and shows overall better diagnostic accuracy [11].

There may be several reasons for these differences among studies. The choice of the optimal cut-off value is to some extent subjective and depends on what ratio between sensitivity and specificity is preferred. Second, the criteria for MCI diagnosis may differ between studies. In addition, age and years of education vary between studies. The original MoCA study recommends adding 1 point for individuals with 12 years of education or less on their total score [6], however, the recommended 1-point correction has been debated as insufficient to compensate for educational differences [23]. The use of age- and education-adjusted norms in order to avoid misdiagnosing cognitive impairment has been proposed by many studies [17, 19, 20, 24].

Despite MoCA being available for a long time, the present study is the first to examine the validity of MoCA for detecting MCI in the Slovenian population. The use of MoCA besides MMSE is recommended in clinical practice. In view of future upcoming disease modifying therapy for Alzheimer’s disease, the use of well-defined cut-off values for the population in the screening process is of additional importance. Optimal cut-off values to detect MCI may be lower than previously recommended and a score of 25/26 points can reduce the number of false positives.

A strength of our study is that MCI was diagnosed based on neuropsychological testing covering cognitive domains of delayed memory, visuospatial abilities, and executive function, with a relatively strict threshold of 1.5 SD below the norm. Furthermore, the MCI group and healthy controls did not differ in terms of age and education, which supports the notion that our results were most likely not influenced by these two factors, but truly reflect differences in cognitive functioning of individuals. An additional strength of our study is a very well-characterised group of MCI with around 41% of MCI subjects having MCI due to AD according to a detailed diagnostic assessment including CSF biomarker analysis. Some limitations of the study must be addressed. At the optimal value of 23/24, both sensitivity and specificity were moderately high, suggesting that the Slovenian MoCA may not be as robust in accurately identifying individuals with MCI. Several factors may contribute to this finding, including cultural differences, patient individual differences (cognitive reserve, motivation, and effort), the heterogeneity of our study population, and even potential interpretation variability. Unfortunately, due to the smaller sample size, we were not able to classify MCI into single/multiple domain and non-amnestic/amnestic groups.

In conclusion, MoCA compared to MMSE had better diagnostic accuracy for detecting MCI in a Slovenian sample. Our study did not replicate the originally recommended cut-off score of 25/26 but instead indicated that a score of 23/24 is more suitable for recognizing individuals with MCI.

## Data Availability

Not applicable.
